# 26 years of mooring data from Olympic Coast National Marine Sanctuary

**DOI:** 10.1016/j.dib.2026.112998

**Published:** 2026-06-20

**Authors:** Brandy T. Cervantes, Kathryn R. Hough, Jeannette Waddell, Melanie R. Fewings, Craig M. Risien, John B. Mickett

**Affiliations:** a College of Earth, Ocean, and Atmospheric Sciences, Oregon State University, 104 CEOAS Admin Building, Corvallis, OR 97331, USA; b Olympic Coast National Marine Sanctuary, 115 East Railroad Ave #301, Port Angeles, WA 98362, USA; c Olympic Coast National Marine Sanctuary (Retired), USA; d Applied Physics Laboratory, University of Washington, 1013 NE 40th St, Seattle, WA 98105, USA

**Keywords:** California current, Washington state, Subsurface, Seawater temperature, Practical salinity, Dissolved oxygen, Water velocity

## Abstract

Olympic Coast National Marine Sanctuary (OCNMS) off Washington State was designated by the National Oceanic and Atmospheric Administration (NOAA) in 1994 and is embedded in the northern California Current (NCC) system, which is affected by climate fluctuations such as marine heat waves, El Niño, hypoxia, ocean acidification, and changes in timing of the spring transition to upwelling conditions. There is a need to better understand the climatological conditions in the Sanctuary, especially considering the presence of four Coastal Treaty Tribes with treaty-protected rights to marine resources on the Olympic Coast. Oceanographic moorings at five cross-shelf lines along the Olympic Coast have measured surface and subsurface water properties from 2000 to present. Measurements focus on the summer upwelling season, when hydrographic conditions fluctuate on the time scale of local wind events and remotely-generated coastal trapped waves, and biogeochemical stressors like hypoxia and ocean acidification tend to worsen. Additionally, temperature sensors deployed at the Teahwhit Head site and, in more recent years, at the Makah Bay site in 42 meters water depth remain in the water throughout the year to measure winter temperatures. This article provides a description of the OCNMS mooring program and our efforts to combine 26 years of water temperature, salinity, dissolved oxygen, pressure, and velocity data into quality-controlled time series available via Zenodo at https://doi.org/10.5281/zenodo.19751759.

Specifications Table

**Table:** 

Subject	Oceanography
Specific subject area	Hydrographic mooring data collected at 15 stations within 20 km of the coast off northern Washington state, USA (47.35°N to 48.33°N)
Type of data	Figure Raw, Processed
Data collection	Instrumentation used for primary data collection varies by site, depth, and year. Primary temperature data were collected by Onset HOBO TidBiT loggers (models TBI32-05+37 and UTBI-001) throughout the water column and by Sea-Bird Electronics (SBE) 39 sensors near the bottom. SBE 37-SM MicroCATs were used to measure primary temperature and salinity near the surface and bottom. SBE 16+ or 19+ SeaCATs were used to measure primary temperature, salinity, and pressure near the bottom. SBE 16+ or 19+ SeaCATs with pumped SBE 43 and SBE 37 SMP-ODO MicroCATs with pumped SBE 63 were used to measure primary temperature, salinity, dissolved oxygen, and pressure near the bottom. SBE 37 SMP-IDO MicroCATs with pumped SBE 43I were used to measure primary temperature, salinity, and dissolved oxygen near the bottom. Falmouth Scientific 2-Dimensional Acoustic Current Meter (2D-ACM) sensors were used to measure primary water velocity near the surface and near the bottom. HOBO U20 Water Level Loggers were used to measure primary pressure near the surface. The secondary data consists of NetCDf files with pre-processed data, quality-controlled hourly data, and record mean values, as well as CSV files with quality-controlled hourly data.
Data source location	Station Name, Latitude, Longitude, Water Depth [m, at Mean Lower Low Water (MLLW)]: *Makah Bay (MB)* MB015, 48.3254°N, 124.6768°W, 15 MB042, 48.3240°N, 124.7354°W, 42 *Cape Alava (CA)* CA015, 48.1663°N, 124.7568°W, 15 CA042, 48.1660°N, 124.8234°W, 42 CA065, 48.1659°N, 124.8949°W, 65 CA100, 48.1650°N, 124.9319°W, 100 *Teahwhit Head (TH)* TH015, 47.8761°N, 124.6195°W, 15 TH042, 47.8762°N, 124.7334°W, 42 TH065, 47.8767°N, 124.7967°W, 65 *Kalaloch (KL)* KL015, 47.6008°N, 124.4284°W, 15 KL027, 47.5946°N, 124.4971°W, 27 KL050, 47.5933°N, 124.6112°W, 50 *Cape Elizabeth (CE)* CE015, 47.3568°N, 124.3481°W, 15 CE042, 47.3531°N, 124.4887°W, 42 CE065, 47.3528°N, 124.5669°W, 65 Primary data source: Olympic Coast National Marine Sanctuary https://olympiccoast.noaa.gov/science/oceanographic-moorings/data.html
Data accessibility	The data described here are available at: Repository name: Zenodo Data identification number: 10.5281/zenodo.19751759 Direct URL to data: https://doi.org/10.5281/zenodo.19751759
Related research article	None

## 1 Value of the Data

• Despite projected warming in Washington coastal waters during the 21st century [1], there are no published investigations of temperature trends within the area encompassed by the boundaries of Olympic Coast National Marine Sanctuary (OCNMS) [2], and few publications involving temperature observations in OCNMS [3,4].
• Subsurface data are important because bottom temperature variability can have unique and dramatic biological impacts on continental shelves [5]. Recent work on the nearby Oregon continental shelf has shown that surface data alone are not sufficient to monitor and understand anomalous warming events in the Northern California Current (NCC) [6].
• The 2008–2019 OCNMS Condition Report [7] identified changing ocean conditions and ocean acidification as the stressors having the biggest impacts on sanctuary waters. To investigate the impacts of ocean acidification on the Dungeness crab fishery, the most valuable commercial fishery on the Washington coast [8], researchers have used empirical relationships with temperature, salinity, and dissolved oxygen as proxy variables to estimate dissolved inorganic carbon and total alkalinity [9].
• Coastal time series spanning multiple decades are necessary to generate accurate climatologies required to identify and quantify anomalous events because long-term temperature trends of the magnitude observed on global scales are more difficult to detect in coastal upwelling regions due to the high variability [6].
• Sanctuary data are currently used to support regional forecasting and prediction efforts in the NCC including the LiveOcean model [10] (https://faculty.washington.edu/pmacc/LO/LiveOcean.html) for short-term forecasts of ocean conditions and the J-SCOPE model [11] (https://www.nanoos.org/​products/j-scope) for seasonal forecasts of ocean conditions and ecosystem habitats.

## 2 Background

The full suite of mooring data from the OCNMS sites have not previously been publicly available in a universally accessible format, nor have they previously been combined into long time series spanning the duration of the mooring program. Wintertime temperature data collected at the Teawhit Head 42-meter mooring in 2009 and 2014–2025 and at the Makah Bay 42-meter mooring in 2023–2025 were not made publicly available prior to this project. Pressure sensor data collected near the surface at the Makah Bay, Teahwhit Head, and Cape Elizabeth 42-meter moorings from 2015-present were not analyzed prior to this project; here, they are used to determine incidents of mooring blowdown that occur during strong currents. Additionally, the work described here provides quality control for the data collected prior to 2010 and generates flags for the raw data collected from 2010–2025 and the hourly-averaged data for the full time series. The quality flags are defined by the U.S. Integrated Ocean Observing System (IOOS) Quality-Assurance/Quality Control of Real-Time Oceanographic Data (QARTOD). Record means of temperature, salinity, dissolved oxygen, pressure, and velocity are also provided to allow data users to easily calculate anomaly time series.

## 3 Data Description

**Fig. 1:**
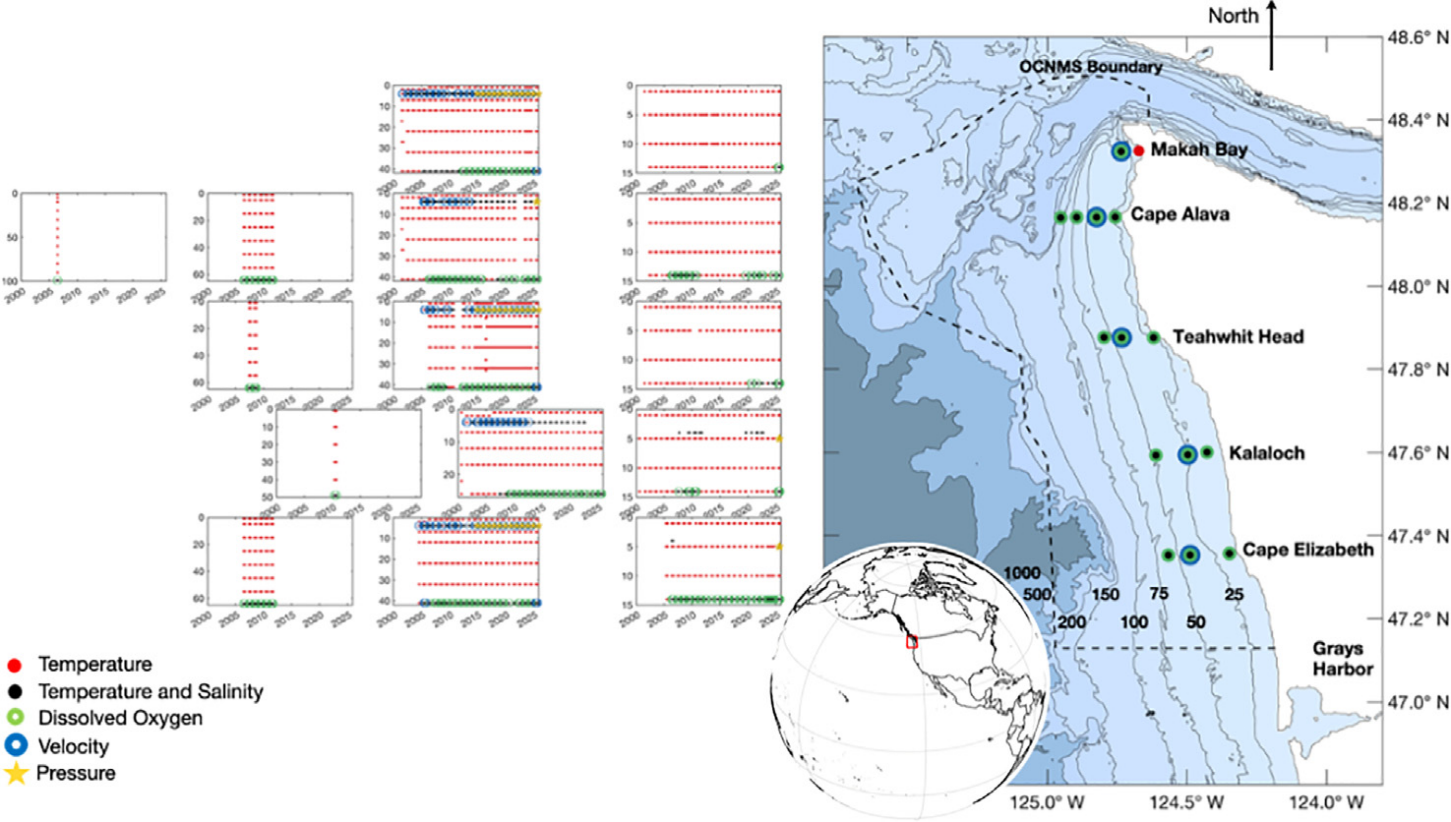
Timeline of measurements made at each OCNMS mooring (left) with placement matching the station locations as shown in the regional map (right) in the longitudinal direction and along isobaths. Timelines that correspond to the 27-meter and 50-meter moorings at Kalaloch are offset from the 42-meter and 65-meter positions. Vertical axis labels indicate nominal instrument depth in meters. See Figs S1-S16 for detailed timelines of instrument types plotted separately for each OCNMS mooring. At right, select isobaths from GEBCO 2022 gridded bathymetry [12] are shown with labels in meters and the OCNMS boundary is indicated by the dashed line. The inset map shows North America with the Washington shelf region indicated as a red box. Legend symbols denote types of data available at each location and depth.

**Fig. 2:**
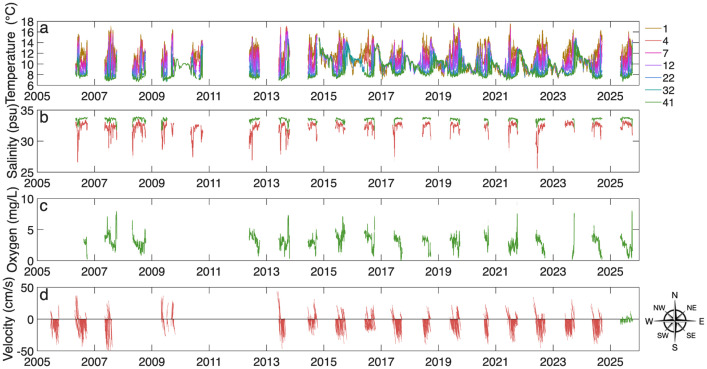
Daily-averaged time series of (a) temperature, (b) salinity, (c) dissolved oxygen, and (d) velocity vectors at all available depths at the Teahwhit Head 42-meter mooring (TH042). Measurement depths in meters are indicated by the colors shown in the legend to the right of panel (a). Gaps in data can occur for individual variables when instruments are not deployed (e.g., oxygen in 2009–2010) or for all variables when a mooring is lost, as TH042 was in 2011. Water velocity magnitude in (d) is indicated by the y-axis and direction is indicated by the compass direction the vector is pointing towards following the oceanographic convention.

**Fig. 3:**
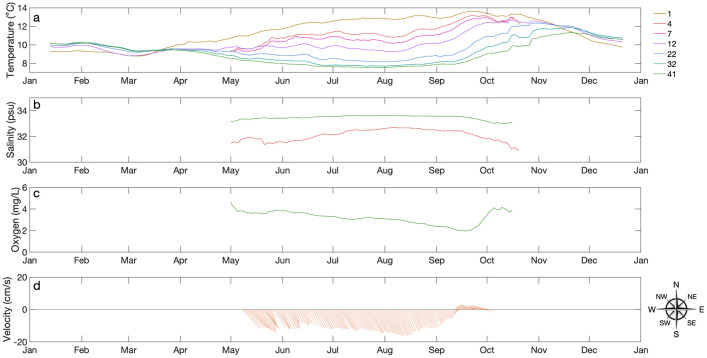
Record means of (a) temperature, (b) salinity, (c) dissolved oxygen, and (d) velocity vectors at all available depths at the Teahwhit Head 42-meter mooring. Measurement depths in meters are indicated by the colors shown in the legend to the right of panel (a). Measurements made by different instruments can have different record mean lengths [e.g., salinity at the near surface and near bottom in panel (b) are measured with different SBE sensors] due to variations in deployment lengths and types of instruments deployed. The years included in the record mean calculation at the Teahwhit Head 42-meter mooring are those with observations shown in Fig. 2 (e.g., 2006–2010 and 2012–2026 for temperature and salinity). This varies by site and variable as described in Section 4.3. Water velocity magnitude in (d) is indicated by the y-axis and direction is indicated by the compass direction the vector is pointing towards following the oceanographic convention.

**Fig. 4:**
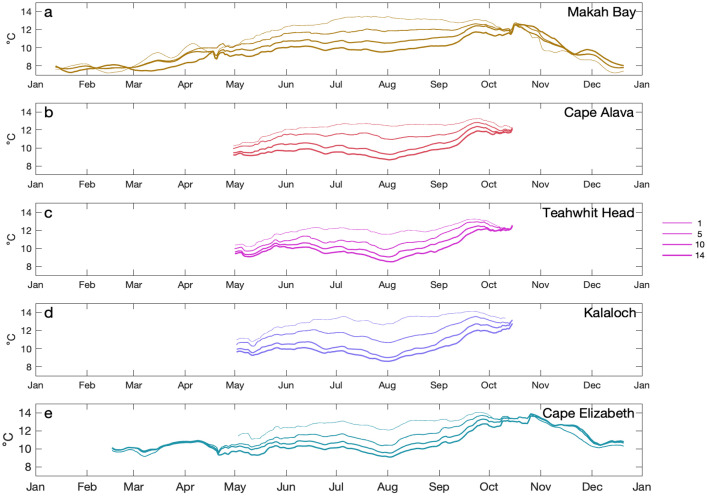
Record means of temperature from the 15-meter sites at (a) Makah Bay, (b) Cape Alava, (c) Teahwhit Head, (d) Kalaloch, and (e) Cape Elizabeth at all available depths. Measurement depths in meters are indicated for all panels by the line thicknesses shown in the legend in panel (c). Note that the winter means at Makah Bay and Cape Elizabeth are based on limited data (2011–2012 and 2013–2014 at Makah Bay and 2023–2024 at Cape Elizabeth).

The oceanographic moorings maintained by OCNMS during 2000-present extend from Cape Elizabeth to Makah Bay at the northwestern edge of Washington (Fig. 1). The temperature, salinity, dissolved oxygen, pressure, and velocity data collected at the OCNMS moorings and the record means as described here are available via Zenodo at https://doi.org/10.5281/zenodo.19751759 [13]. The dataset includes multiple NetCDF files for each mooring site that follow CF (Climate and Forecast) metadata conventions. The site name convention is the geographic location abbreviation followed by the water depth, in meters, at the mooring (Makah Bay: MB015 and MB042; Cape Alava: CA015, CA042, CA065, and CA100; Teahwhit Head: TH015, TH042, and TH065; Kalaloch: KL015, KL027, and KL050; Cape Elizabeth: CE015, CE042, and CE065). *sitename_processed.nc* files contain hourly-averaged temperature, salinity, dissolved oxygen, eastward velocity, northward velocity, and pressure and the corresponding quality flags for each variable, at all available depths from 2000 to 2025 (Fig. 2). While OCNMS refers to the vertical instrument position in terms of altitude from the bottom, this dataset uses the nominal depth (defined as water depth at the site minus instrument altitude). *sitename_record_means.nc* files contain record means calculated as described in Section 4.3 for temperature, salinity, dissolved oxygen, eastward velocity, northward velocity, and pressure using all available years of data for each variable at each measurement depth (Fig. 3, Fig. 4). The record means allow users to calculate anomaly time series for a period of interest by subtracting the climatological values from daily-averaged time series. *sitename_raw_variable_depth.nc* files contain the data at each available depth and mooring site before computing averages and applying quality flags. *sitename_variable.csv* are plain-text ASCII comma-separated value files for each variable at each mooring containing all hourly values with “pass” or “suspect” flags.

**Fig. 5:**
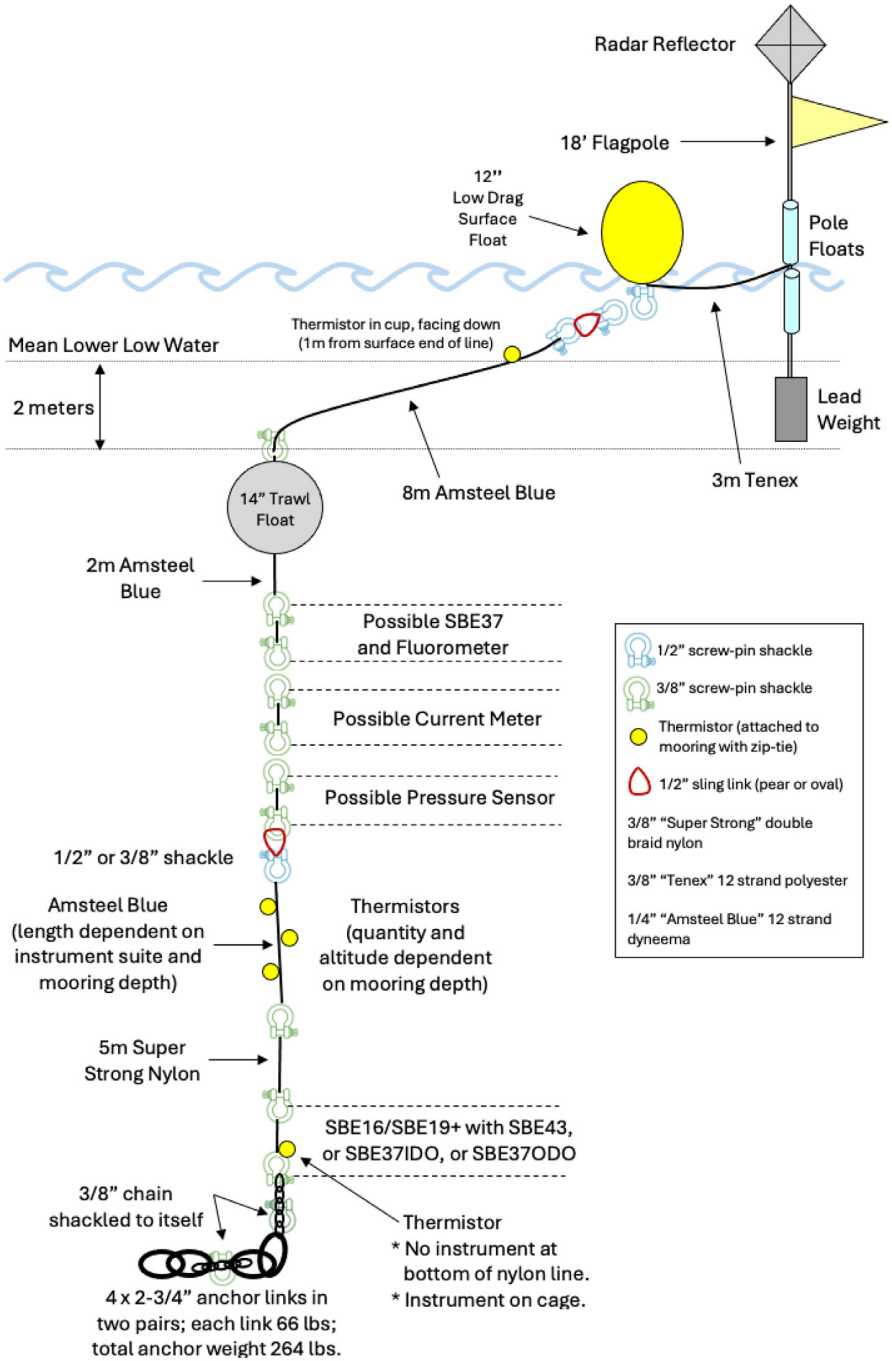
OCNMS mooring diagram. Instruments are mounted at various depths on the moorings, depending on the depth at the site. Mooring hardware size and line diameter is in inches, denoted as " in diagram and legend.

The moorings collect data from late spring to late fall at five locations on the 15-meter isobath and five locations on the 27- or 42-meter isobath (the Kalaloch mid-depth mooring is at 27 m water depth). Moorings have also been deployed at 50 m at Kalaloch; 65 m at Cape Alava, Teahwhit Head, and Cape Elizabeth; and 100 m at Cape Alava during some years. Temperature measurements occur at 5–10 m intervals throughout the water column at all locations, while the other variables are measured near the surface and/or bottom, depending on the variable and the site. OCNMS first deployed moorings in 2000 and as of summer 2025 is maintaining ten moorings spread across the 15-meter sites, the 42-meter sites, and the 27-meter site at Kalaloch. The moorings have a lightweight design (Fig. 5) and are deployed in the spring and recovered in the fall. A few times during the summer, each mooring is recovered for cleaning, servicing, and data download before being re-deployed in the same location.

## 4 Experimental Design, Materials and Methods

### 4.1 OCNMS moorings

All OCNMS moorings measure temperature and several also measure salinity, dissolved oxygen, water velocity, near-surface pressure, bottom pressure, and chlorophyll concentration (chlorophyll is not included in the dataset). The program has used various instrument types over the years (Figs. S1-S16). Temperature is measured throughout the water column every 2, 8, or 10 min, depending on the year and site, with Onset HOBO TidbiT TBI32 -05+37 or UTBI-001 temperature loggers, which have a response time of 5 min in water. Beginning in 2019, temperature is also measured 1 m above the bottom every 2 or 10 min with Sea-Bird Electronics (SBE) 39 temperature recorders. Temperature and salinity are measured 4 m below the surface [i.e., Mean Lower Low Water (MLLW)] and 1 m above the bottom every 1, 2, or 10 min with SBE 37-SM MicroCAT instruments. Starting in 2012, temperature, salinity, and dissolved oxygen are measured 1 m above the bottom every 10 min with SBE 37 SMP-IDO MicroCAT instruments. Temperature, salinity, pressure, and dissolved oxygen are recorded 1 m above the bottom as 4-second averages every 10 min using SBE 16+ or 19+ SeaCAT instruments with pumped SBE 43. In 2019, SBE 37 SMP-ODO MicroCAT instruments with pumped SBE 63 optical dissolved oxygen were added to the instrument fleet. Anti-foulant devices are installed on each end of the SBE conductivity cells to treat any water that enters the cells. Approximately 38 m above the bottom at the 42-meter sites and 23 m above the bottom at the Kalaloch 27-meter site, velocity and temperature are recorded as a 2-minute average of 1-Hz samples every 30 min using Falmouth Scientific 2D-ACM sensors. In 2025, the current meters were moved to 1 m above the bottom. HOBO U20 Water Level Loggers were deployed approximately 0.5 m below the current meters beginning in 2015 to measure pressure near the surface every 30 min at the Makah Bay, Teahwhit Head, and Cape Elizabeth 42-meter sites (Fig. 1). The pressure sensors continued to be deployed near the surface at the Makah Bay, Teahwhit Head, and Cape Elizabeth 42-meter sites for the first deployment in 2025, then they were moved to the Cape Alava 42-meter, Kalaloch 15-meter, and Cape Elizabeth 15-meter sites for the remaining 2025 deployments.

### 4.2 Data processing

**Fig. 6:**
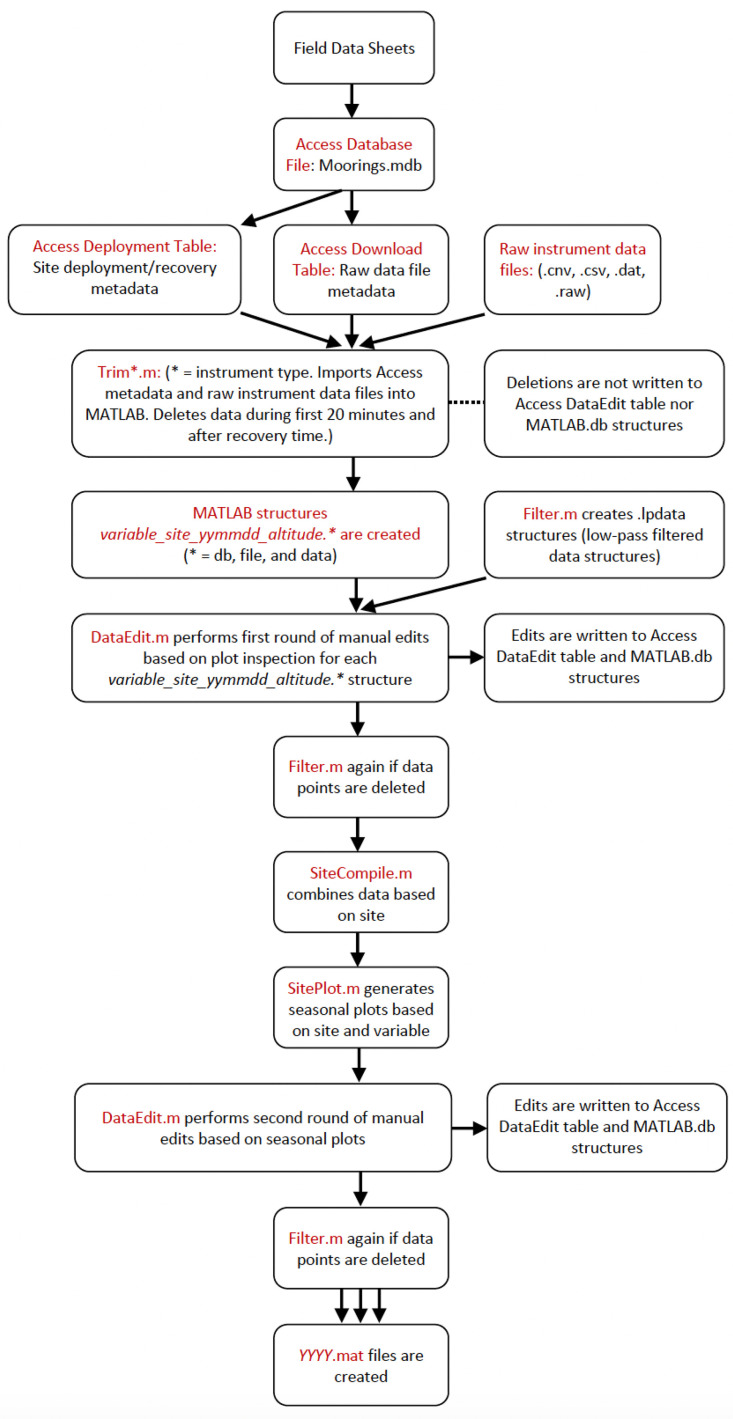
Flowchart of mooring data processing steps taken by OCNMS staff after raw data are downloaded throughout the field season, beginning with Microsoft Access database files and ending with MATLAB^Ⓡ^ files containing all the data for each season. The altitude used in the MATLAB^Ⓡ^ structure names refers to the distance between the anchor and the instrument.

Sanctuary staff collect raw instrument downloads during the field season and process SBE and HOBO data files using SBE and Onset software, respectively, to generate ASCII text files. Below, we refer to these as the “pre-processed” data. The text files are then loaded into MATLAB^Ⓡ^ and bad data are annotated and removed by a combination of automatic and subjective (e.g. identifying periods when instruments are out of the water during deployment and recovery), criteria to create an individual MATLAB^Ⓡ^ structure for each instrument and each deployment, which are then grouped by deployment year, named as *YYYY.mat* (Fig. 6), compressed into ZIP files, and made available on the OCNMS website (https://olympiccoast.noaa.gov/science/oceanographic-moorings/data.html). Below, we refer to these as the “processed” data.

**Fig. 7:**
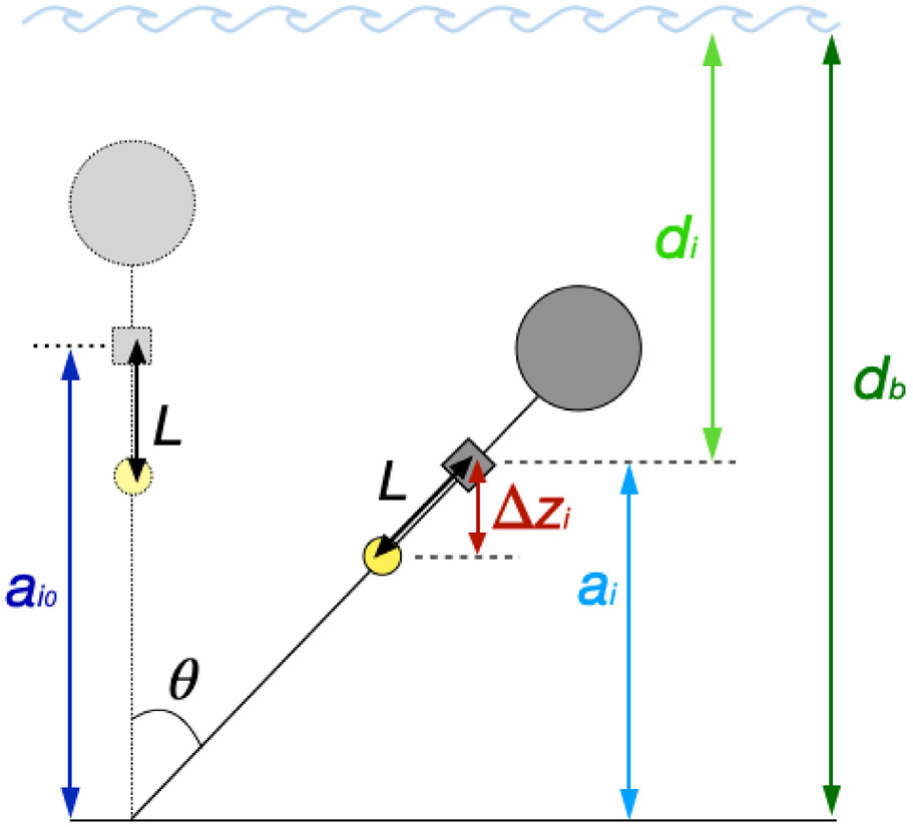
Schematic of mooring blowdown event as discussed in Section 4.2, showing the pre-blowdown mooring position in light gray and the post-blowdown position in dark gray. The gray circle represents the trawl float, the gray square represents the instrument cluster of the 4-m temperature sensor, current meter, and pressure sensor, and the yellow circle represents the 12-meter temperature sensor. Symbols are defined in Section 4.2.

Working from the thousands of MATLAB^Ⓡ^ structures containing the processed data, we created output files in user-friendly formats that include the complete time series for all variables at all locations on a common, hourly-averaged timebase. The workflow for creating the datasets described here is as follows:

1. Check data collected prior to 2010, during which time the pre-processed data files are not available, and remove bad points using both automated and subjective criteria similar to those used by OCNMS. In the case of dissolved oxygen, we used feedback from experts to remove points that were not realistic (Simone Alin, *personal communication*) and we used a combination of subjective and automated methods to remove patches of extremely noisy data observed at various times and locations. To remove occasional outliers in salinity and dissolved oxygen, we applied a moving median filter with a 36-hour window and a threshold of 5 multiples of the median absolute deviation.
2. For 2010 and later, compare pre-processed and processed data point-by-point and generate flags for the pre-processed time series (i.e. points that are not equivalent between the two datasets will be assigned a flag value of 3 or 4). The quality flags follow QARTOD standards with 1 = pass, 2 = not evaluated, 3 = suspect or of high interest, 4 = fail, and 9 = missing.
3. When duplicate measurements are available, retain the data from more reliable instruments; e.g., SBE temperature sensors (Fig. 9) are more accurate than TidbiT loggers (see Limitations section below).
4. Using the hourly near-surface pressure, bottom pressure, and near-surface temperature data during 2015–2025, identify as incidents of mooring blowdown (Figs. 7,8) periods when the change in altitude (a) of the near-surface pressure sensor, Δa, due to blowdown is large enough to cause a change in temperature greater than 1 °C, as in Eqn. (1).
  (1) $$\frac{\Delta T}{\Delta z_{i}}\cdot d_{i}>1{░}^{\circ }C$$

**Fig. 8:**
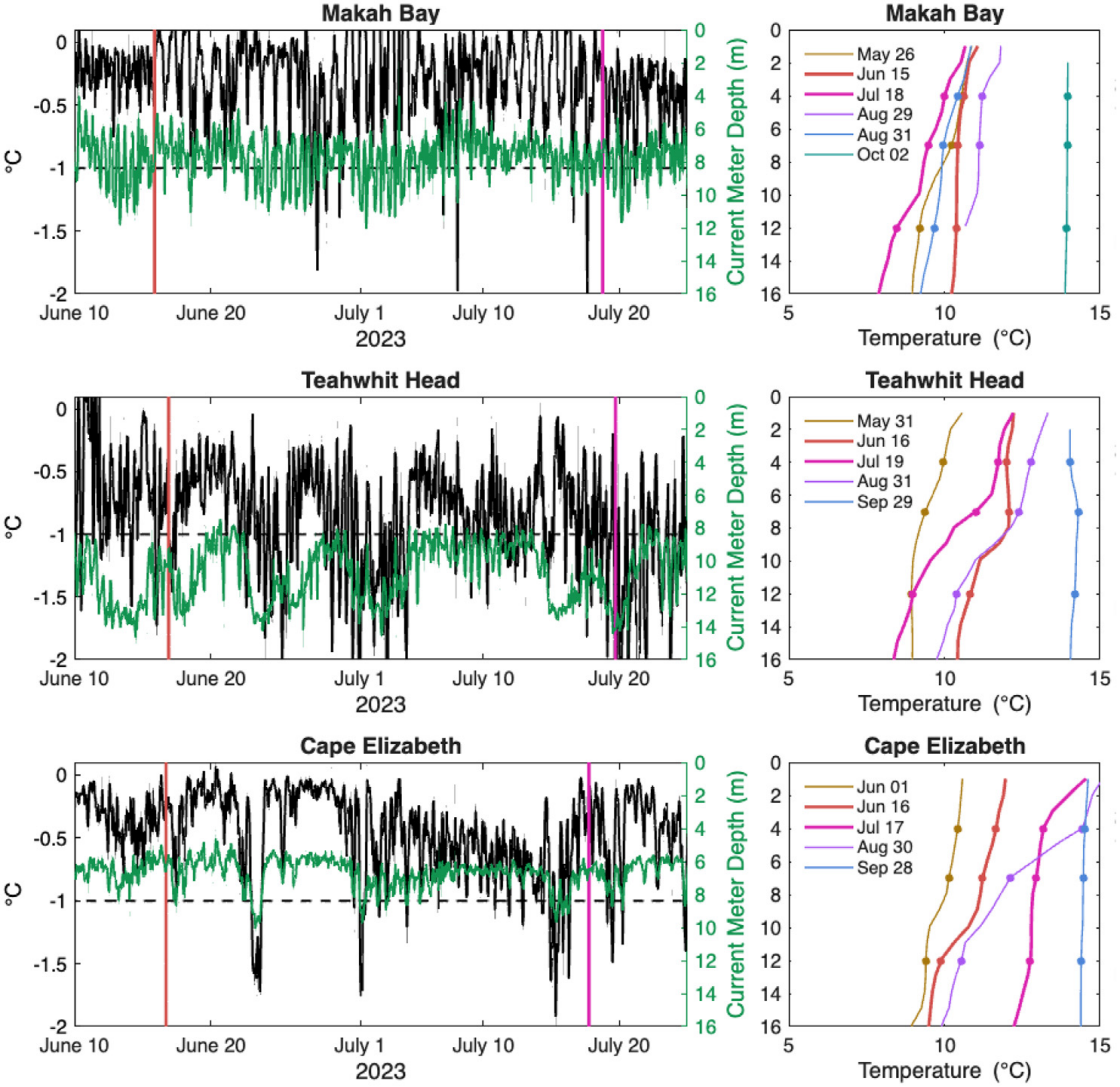
(Left column) Hourly current meter depth (green line; right axis) and $\frac{\Delta T}{\Delta z_{i}}\times d_{i}$ (Eqn. (1)) (black line; left axis) from June 10 – July 25, 2023 at the 42-meter isobath sites at Makah Bay (top row), Teahwhit Head (middle row), and Cape Elizabeth (bottom row). Horizontal dashed line indicates the threshold for flagging blowdown events. Dates of temperature profiles shown in the right column are indicated by the matching colored vertical lines. (Right column) Temperature profiles from the shipboard CTD at the dates shown in the legend during the 2023 deployment season with thick lines indicating the profiles that overlap with the time period shown in the left column (June 16 and July 17, 2023). Nominal depths of moored temperature measurements (4, 7, and 12 m) are shown by the filled circles.
  where ΔT is the difference in temperature at the 4-meter and 12-meter temperature sensors, $d_{i}$ is the depth of the near-surface pressure sensor, and $\Delta z_{i}$ is the distance between the 4-meter and 12-meter temperature sensors after blowdown as in Eqn. (4). The change in altitude, Δa, of the pressure sensor due to blowdown is
  (2) $$\Delta a\;=\;a_{i}-a_{i0}$$
  where $a_{i0}$ is the nominal altitude of the pressure sensor, and the time-varying altitude $a_{i}$ is
  (3) $$a_{i}=d_{b}-d_{i}$$
  where $d_{b}$ is the depth at the seafloor. The time-varying $d_{i}$ and $d_{b}$ are known from the near-surface pressure sensor mounted below the current meter and 4-m temperature sensor and from the bottom pressure sensor, respectively. The distance between the 4-meter and 12-meter temperature sensors after blowdown is
  (4) $$\Delta z_{i}=cos\left(\theta \right)\cdot L$$
  where L is the 8-meter length of the mooring line between the 4-meter and 12-meter temperature sensors and the blowdown angle θ is
  (5) $$\theta =cos^{-1}\frac{a_{i}}{a_{i0}}$$
  We flag as suspect the processed near-surface data (near-surface velocity, near-surface salinity, and temperature data within 12 m of the surface) that meet this criterion. Further description of the hydrostatic depth calculation is provided below. See Fig. 7 for a schematic representation of a blowdown event.
5. To identify mooring blowdown periods prior to the availability of near-surface pressure sensor data in 2015, perform a linear regression, $d_{i}$*= mx + B*, where *x* is the time-varying current meter tilt during 2015–2024 (the 2025 data are omitted from this regression because the current meters are located near the bottom in 2025 instead of adjacent to the pressure sensors as in previous years). The correlation coefficient between pressure sensor depth and current meter tilt is *r^2^ =* 0.53 at Makah Bay, *r^2^ =* 0.77 at Teahwhit Head, and *r^2^ =* 0.72 at Cape Elizabeth. Replace $d_{i}$ with the least-squares fit, *mx + B*, where *x* is the current meter tilt during 2005–2014, in Eqn. (1) and flag as suspect the processed near-surface velocity, near-surface salinity, and temperature data within 12 m of the surface that meet this criterion.
6. Compute hourly-averaged values and record means for all processed data.
7. Organize all data fields (pre-processed data and flags, hourly-averaged processed data and flags, and derived record means) as MATLAB^Ⓡ^ structures corresponding to each mooring site with each variable having dimensions of depth x time.
8. Generate NetCDF and CSV files for each site as described in Section 3.

Absolute pressure, *p_abs_*, measured by the near-surface pressure sensors and bottom pressure sensors was used to calculate hydrostatic depth, *d* (Eqn. (6))

(6) $$d\;=\;\frac{\;p_{abs}-\;p_{atm}}{\rho g}$$

where ρ is water density and g is the acceleration due to gravity (9.8 m/s^2^). The atmospheric pressure, $p_{atm}$, was measured by National Data Buoy Center (NDBC) station 46041, with the National Science Foundation Ocean Observatories Initiative (OOI) Coastal Endurance Array Washington Shelf site meteorological buoy filling in during short periods when 46041 was unavailable. Density (ρ) was calculated using the in-situ temperature and salinity from the SBE 37-SM MicroCAT instruments with the Gibbs Seawater Oceanographic Toolbox for MATLAB^Ⓡ^ based on the Thermodynamic Equation of Seawater 2010 (TEOS-10).

We use the criterion in Eqn. (1) to identify blowdown events because when the near-surface sensors are pushed below the surface mixed layer by strong currents, variability assumed to be caused by horizontal advection will be biased by vertical variability, and this criterion provides an estimate of the level of contamination in units of ΔT. When available, temperature profiles from the shipboard CTD [4] provide snapshots with higher vertical resolution than the moored temperature sensors for an additional check of the temperature gradient (Fig. 8). Based on this criterion, blowdown events were identified at the 42-meter moorings at Makah Bay, Teahwhit Head, and Cape Elizabeth from 2015–2025 with substantial variability in the number of events between sites. Makah Bay experiences blowdown events 3% of the time and occurrence increases to 32% at Teahwhit Head and 15% at Cape Elizabeth. We include the criterion $\frac{\Delta T}{\Delta z_{i}}\cdot d_{i}$ from Eqn. (1) and the instrument altitude change Δa from Eqn. (2) along with the flags and hourly-averaged data at the Makah Bay, Teahwhit Head, and Cape Elizabeth 42-meter sites so users who prefer a different threshold for blowdown events may use these diagnostics to flag the data themselves.

Additionally, velocity data were initially corrected by OCNMS for magnetic declination using a location midway between the Cape Elizabeth and Makah Bay 42-meter moorings (47.84°N, 124.61°W) using a date in the middle of the deployment season for each year. As part of the velocity data quality control process, we removed the original correction and applied a correction calculated at each 42-meter mooring location on a date chosen to be representative of mid-deployment season (July 21) using the International Geomagnetic Reference Field (IGRF) model (https://www.ngdc.noaa.gov/geomag/calculators/magcalc.shtml). Using the magnetic declination correction at each mooring location compared to the midway correction changes the value by 0.8–1.2%, depending on the year and location.

### 4.3 Record mean calculation

The record mean for each variable at each available depth, *X_m_*, is computed similarly to the climatology method in [14] as the mean at yearday *j* using all data within an 11-day window as

(7) $$X_{m}=\sum_{y=y_{s}}^{y_{e}}\sum_{d=j-5}^{j+5}\frac{X\left(y,d\right)}{11\left(y_{e}-y_{s}+1\right)}$$

where *d* is the day of year, *y_s_* and *y_e_* are the starting and ending year of the mean period, and *X(y,d)* is the value on day *d* of year *y*, such that January 1 is day 1*.* Due to the variability in timing of deployments from year to year, a smaller number of observations are available at the start and end of the season. Thus, to remove these end segments with incomplete data availability, we compute *X_m_* only when the 11-day window is filled with elements of *X(y,d)*. While the calculation is the same for all variables and all sites, record means at some sites are based on only 1–3 years of data, and record means at other sites have up to 25 years of data (see Fig. 1). We consider the mean to approximate a climatology if the record includes 15 or more years of data (i.e., temperature, salinity, dissolved oxygen, and velocity at TH042 and CE042; temperature, salinity, and dissolved oxygen at KL027; temperature, salinity, and velocity at MB042; temperature and salinity at CA042; temperature at all five 15-m sites). Only record means computed over the primary spring-fall sampling season can be considered to approximate a climatology as the winter records typically only exist for a few years (see Figs. S2, S3, S9, and S14).

## Limitations

**Fig. 9:**
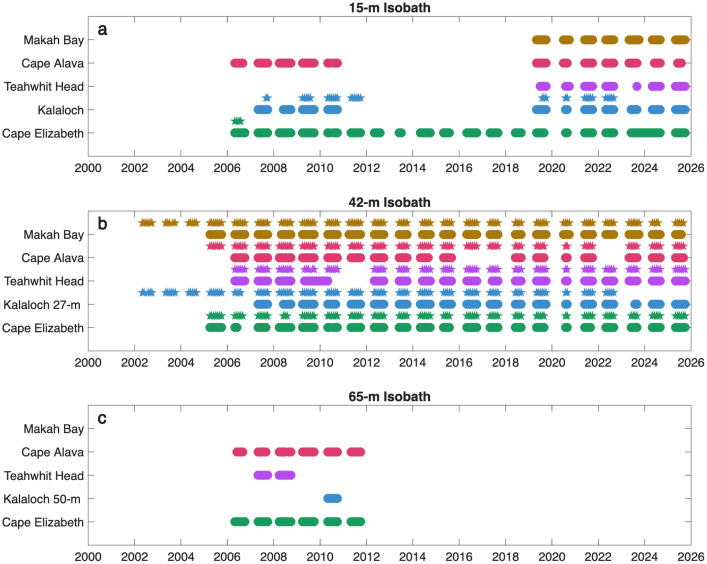
Availability of Seabird (SBE) temperature measurements at the (a) 15-meter sites, (b) 42-meter sites and Kalaloch 27-meter site, and (c) 65-meter sites and Kalaloch 50-meter site, at the near bottom (circles) and near surface (stars), with colors corresponding to the mooring locations as in Fig. 2, Fig. 3, Fig. 4. The Cape Alava 100-m site, which is omitted from this figure, includes SBE temperature data in June-July 2006.

The Onset HOBO TidbiT loggers, which record temperature at depths between the surface and bottom SBE sensors, are less accurate than the SBE sensors. Availability of the SBE sensors varies by year and site (Fig. 9). The manufacturer's stated accuracy of TidbiT loggers is 2 orders of magnitude less than the SBE temperature sensors (TBI32 -05+37 and UTBI-001: 0.2 °C, SBE sensors: 0.002 °C).

Additionally, the OCNMS moorings experience blowdown events during which instruments can be pushed up to 20 m deeper than the intended measurement depth in extremely strong currents. This issue may impact the interpretation of the spatial and temporal variability in the temperature, salinity, and velocity data in the top ∼12 m during the identified blowdown events because the downward vertical movement of the sensors can produce a signal that would appear to be caused by horizontal advection or other mechanisms.

Raw data files and metadata are unavailable prior to 2010, so flags for these years could not be produced by comparing data points between the raw and processed data. Therefore, the processed data from 2000–2009 were manually checked for any large spikes, noisy periods, or unreasonable values, which were then removed and flags were assigned to the hourly-averaged values.

## CRediT Author Statement

**Brandy T. Cervantes**: Conceptualization, Funding Acquisition, Project administration, Formal analysis, Visualization, Data Curation, Software, Validation, Methodology, Writing – Original Draft, Writing – Review and Editing; **Kathryn R. Hough**: Investigation, Resources, Data Curation, Software, Validation, Writing – Review & Editing; **Jeannette Waddell**: Conceptualization, Funding Acquisition, Investigation, Resources, Data Curation, Methodology; **Melanie R. Fewings**: Conceptualization, Funding Acquisition, Project administration, Supervision, Methodology, Writing – Review & Editing; **Craig M. Risien**: Conceptualization, Funding Acquisition, Methodology, Writing – Review & Editing; **John B. Mickett**: Data Curation, Methodology, Writing – Review & Editing.

## Ethics Statement

The authors confirm that they have read and followed the ethical requirements for publication in Data in Brief. The work described in this paper did not involve human subjects, animal experiments, nor data collected from social media platforms.

The scientific results and conclusions, as well as any views or opinions expressed herein, are those of the authors and do not necessarily reflect the views of NOAA or the Department of Commerce. This publication does not constitute an endorsement of any commercial product or intend to be an opinion beyond scientific or other results obtained by the National Oceanic and Atmospheric Administration (NOAA).

## Data Availability

Zenodo26 Years of Mooring Data from Olympic Coast National Marine Sanctuary (Original data).

## Appendix Supplementary materials

Image, application 1
