# Supplementary material for: 26 years of mooring data from Olympic Coast National Marine Sanctuary
Source: Data Brief. 2026 Jun 20;67:112998. doi: 10.1016/j.dib.2026.112998 (PMC13320424; doi:10.1016/j.dib.2026.112998)
Supplement: Supplementary file 1 [file mmc1.docx]

### Supplementary Figures


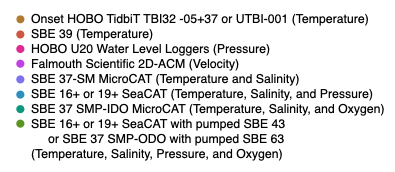


Figure S1. Legend for Figures S2-S16 indicating the instruments used on the OCNMS moorings with the variables they measure in parentheses.


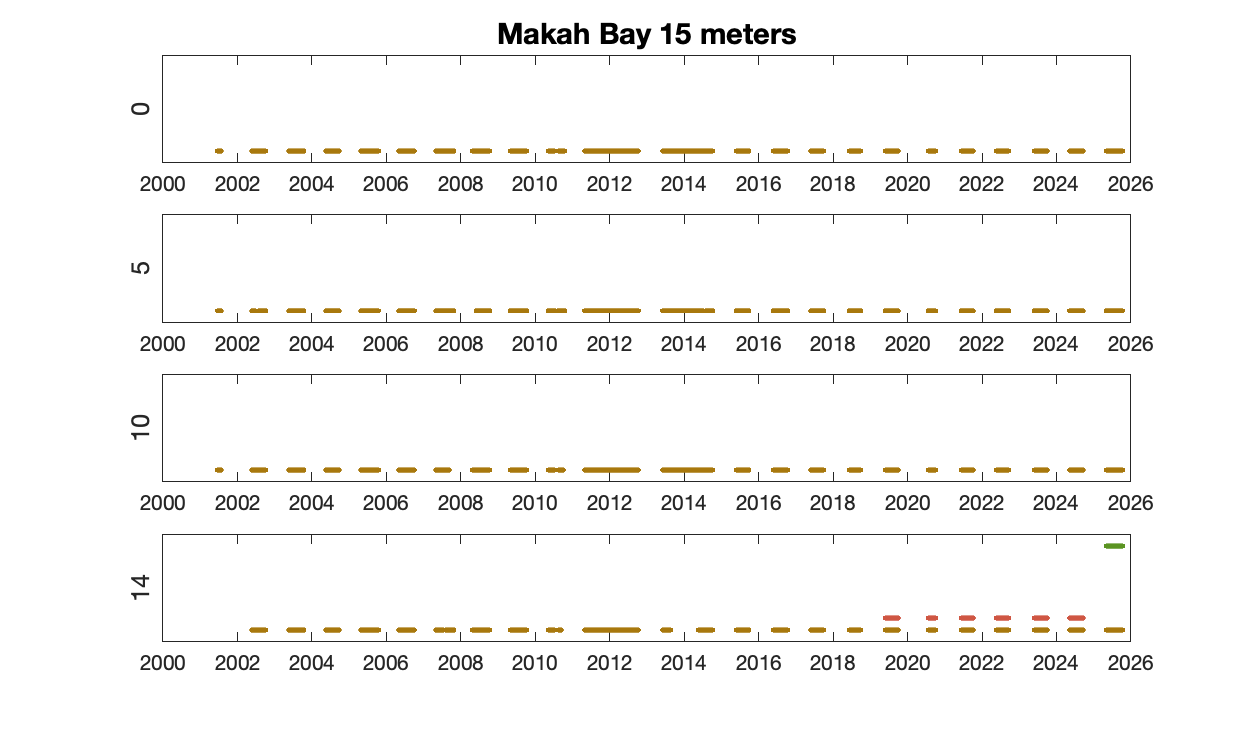


Figure S2. Timeline of measurements at the Makah Bay 15-meter isobath site. For Figs. S2-S16, the instrument types are shown in Fig. S1 and the vertical axis labels show the nominal instrument depth in meters.


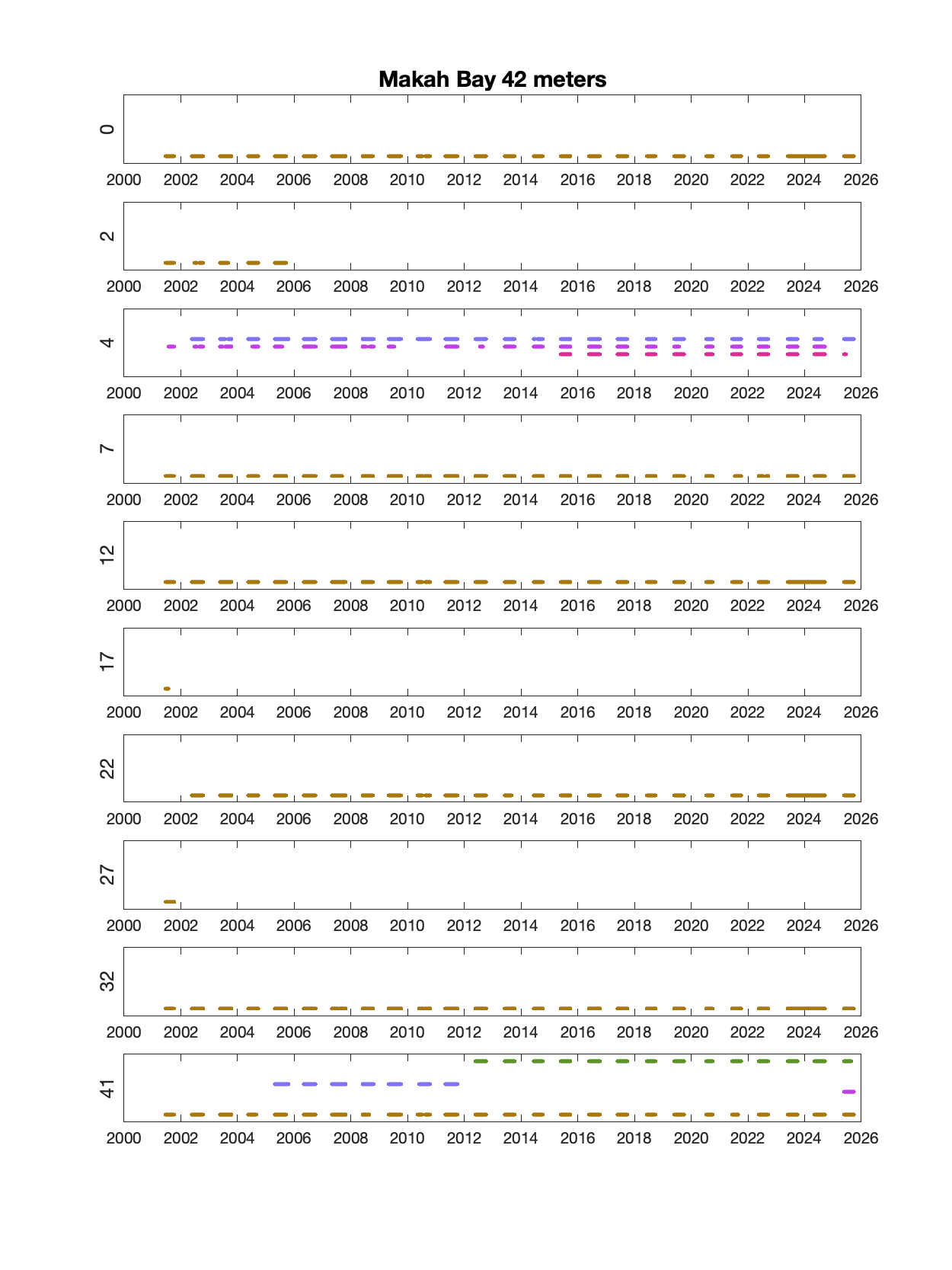


Figure S3. Timeline of measurements at the Makah Bay 42-meter isobath site.


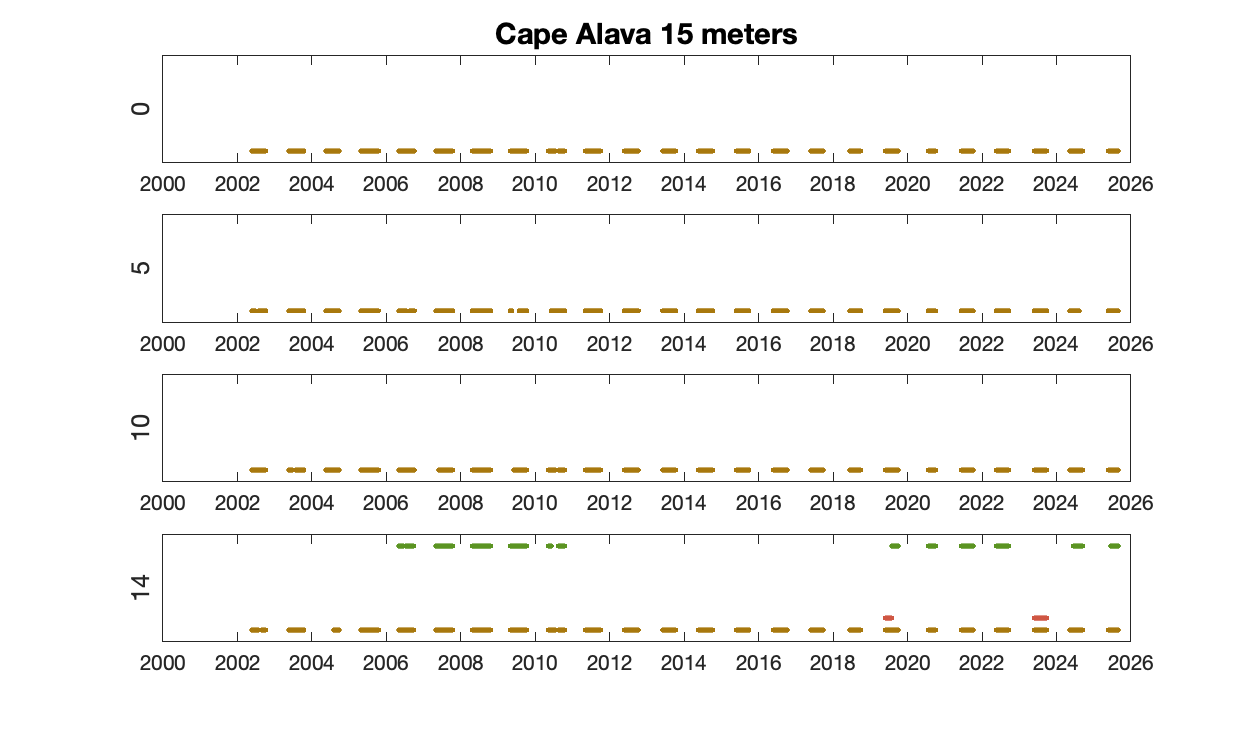


Figure S4. Timeline of measurements at the Cape Alava 15-meter isobath site.


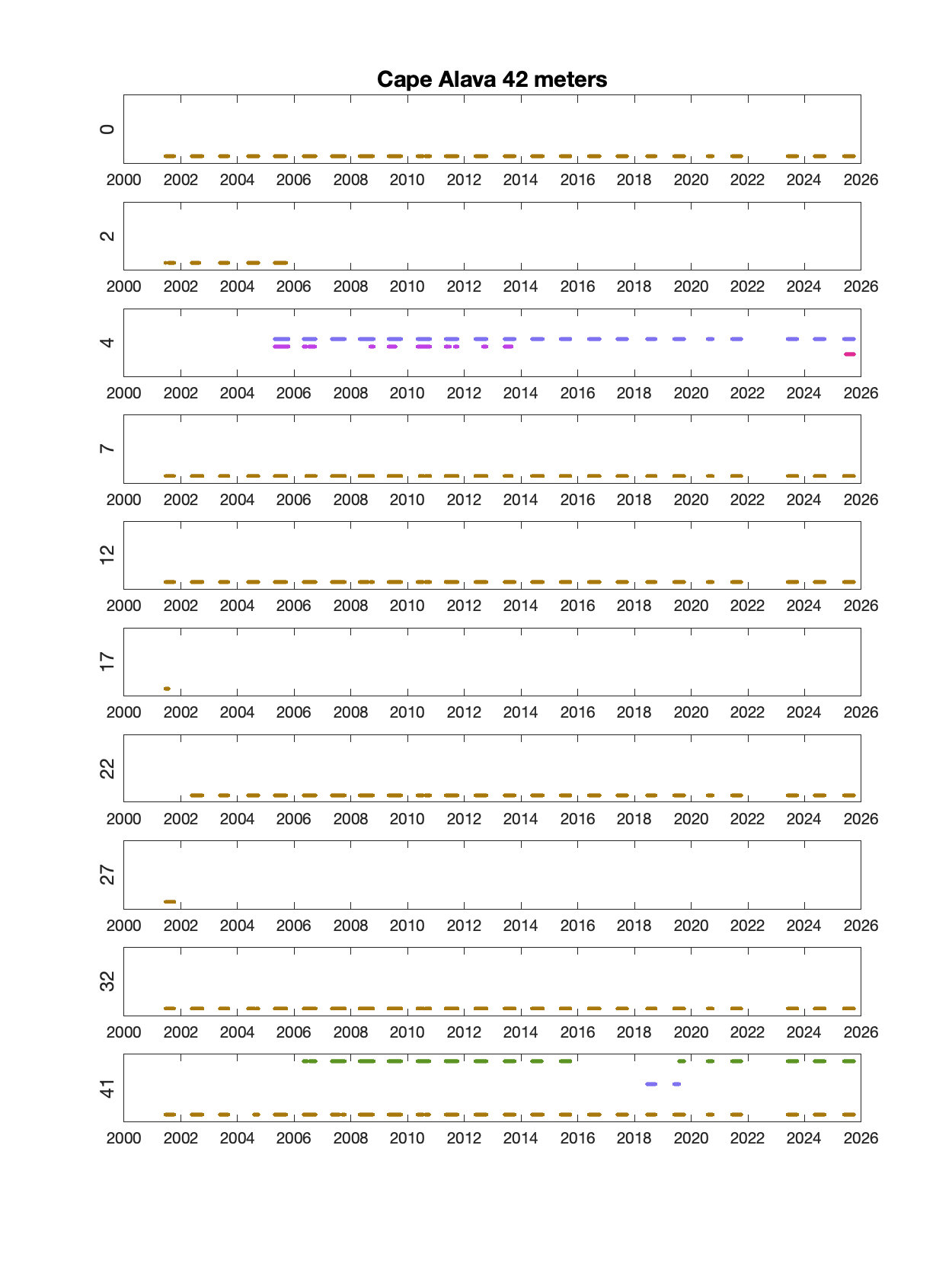


Figure S5. Timeline of measurements at the Cape Alava 42-meter isobath site.


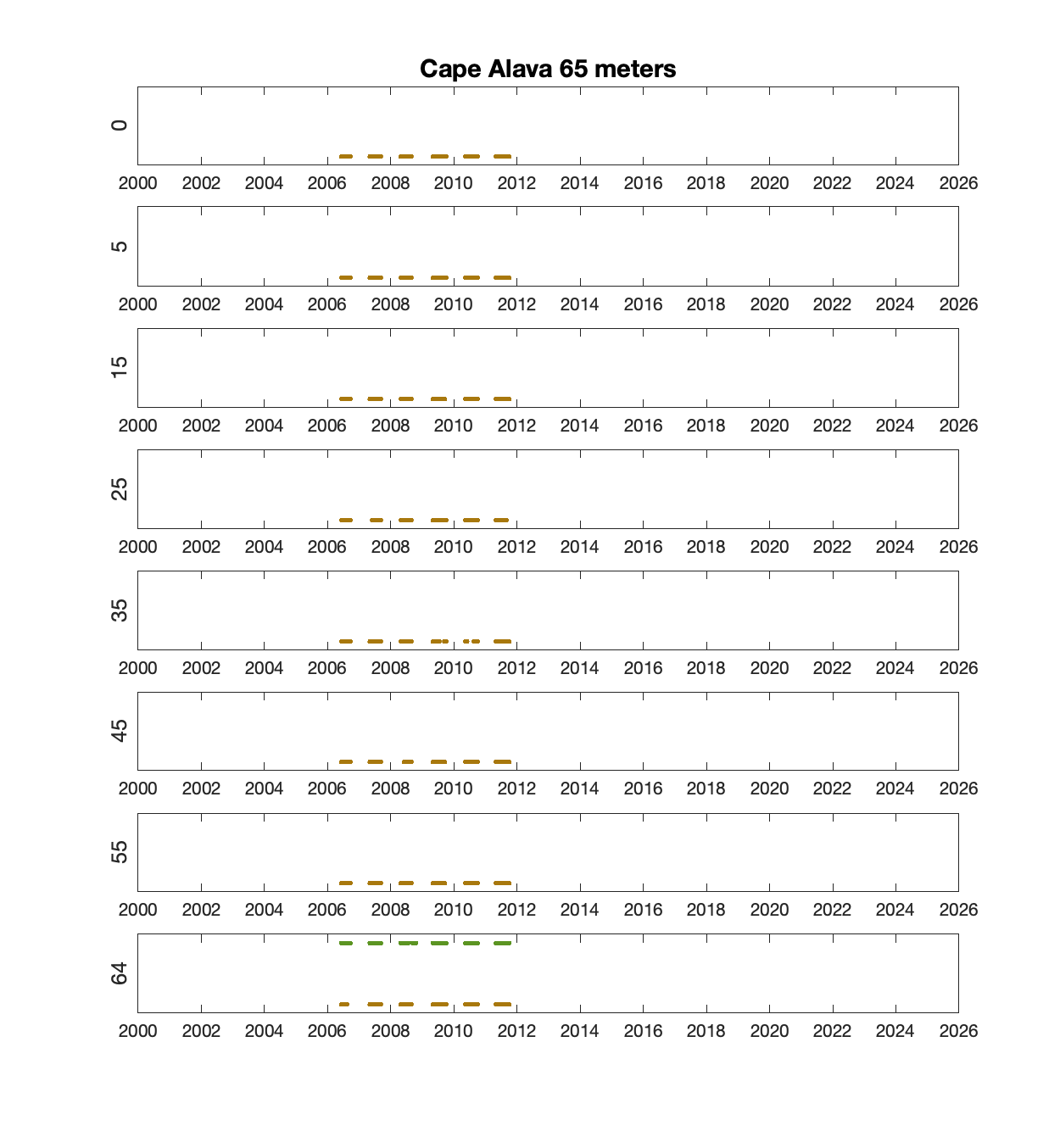


Figure S6. Timeline of measurements at the Cape Alava 65-meter isobath site.


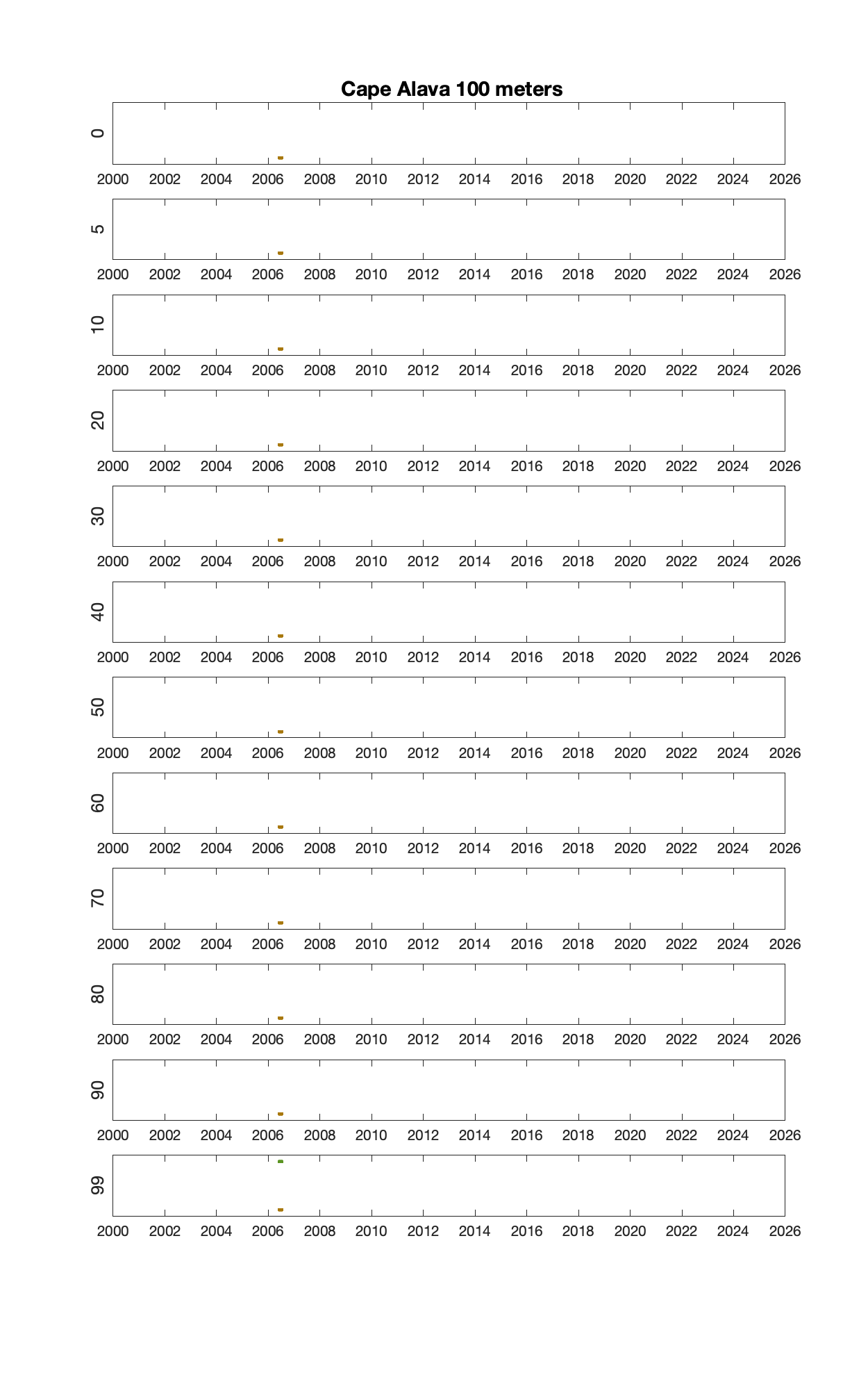


Figure S7. Timeline of measurements at the Cape Alava 100-meter isobath site.


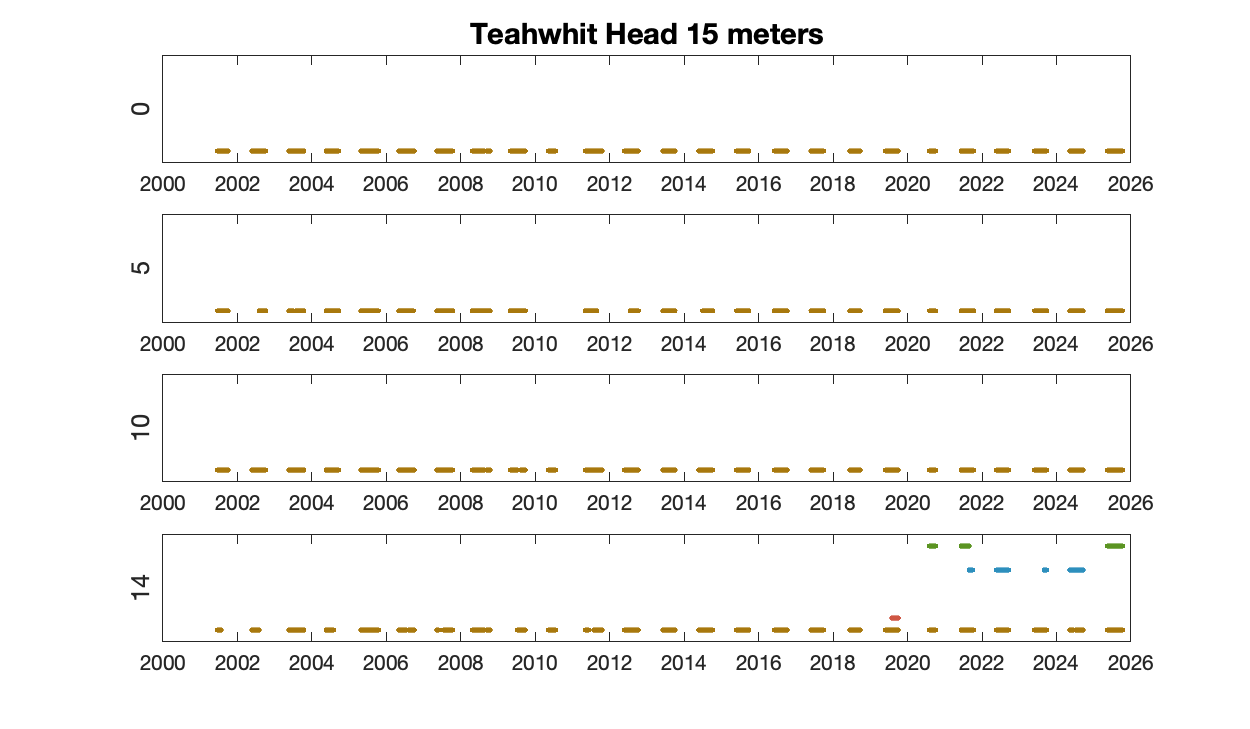


Figure S8. Timeline of measurements at the Teahwhit Head 15-meter isobath site.


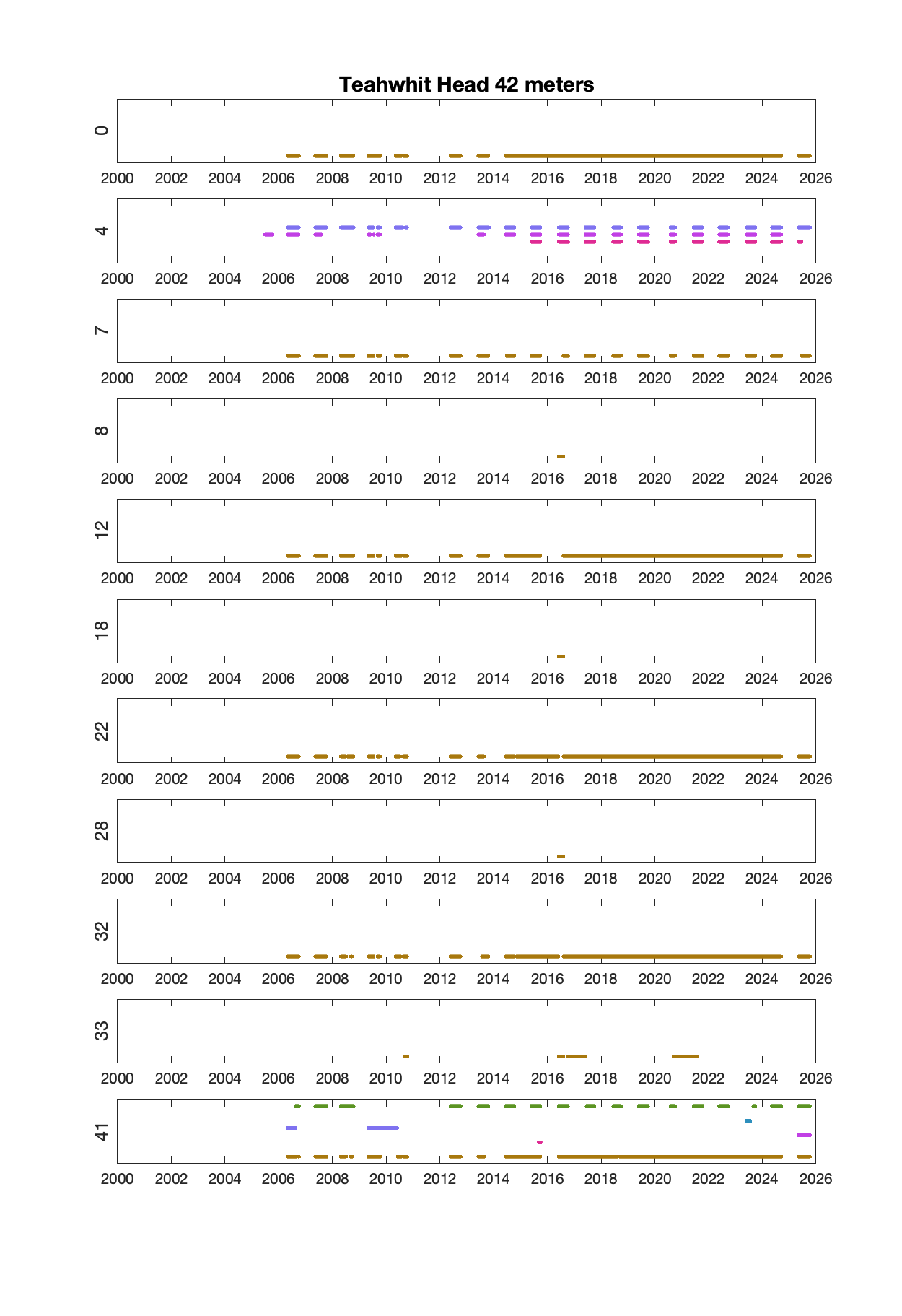


Figure S9. Timeline of measurements at the Teahwhit Head 42-meter isobath site.


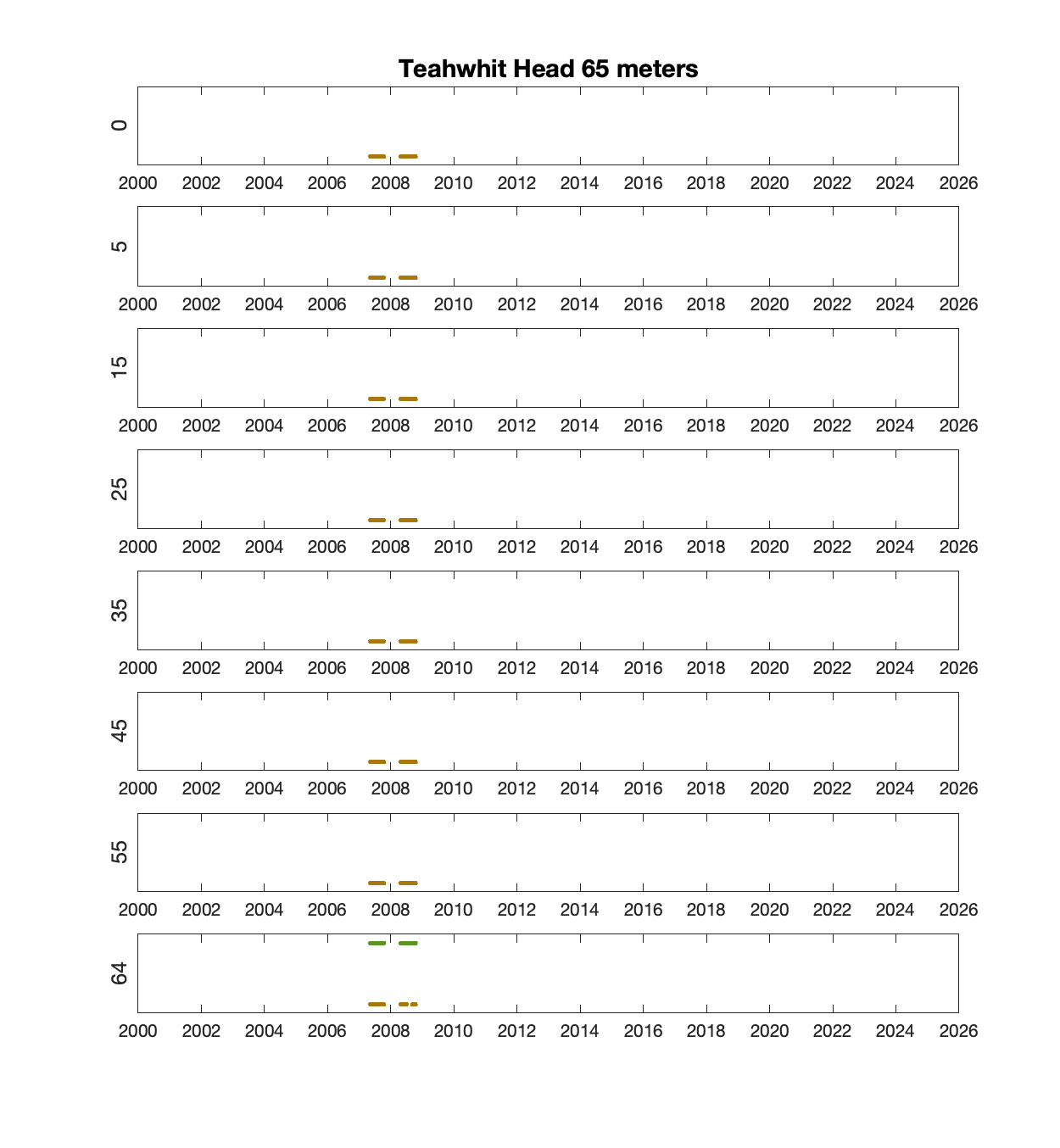


Figure S10. Timeline of measurements at the Teahwhit Head 65-meter isobath site.


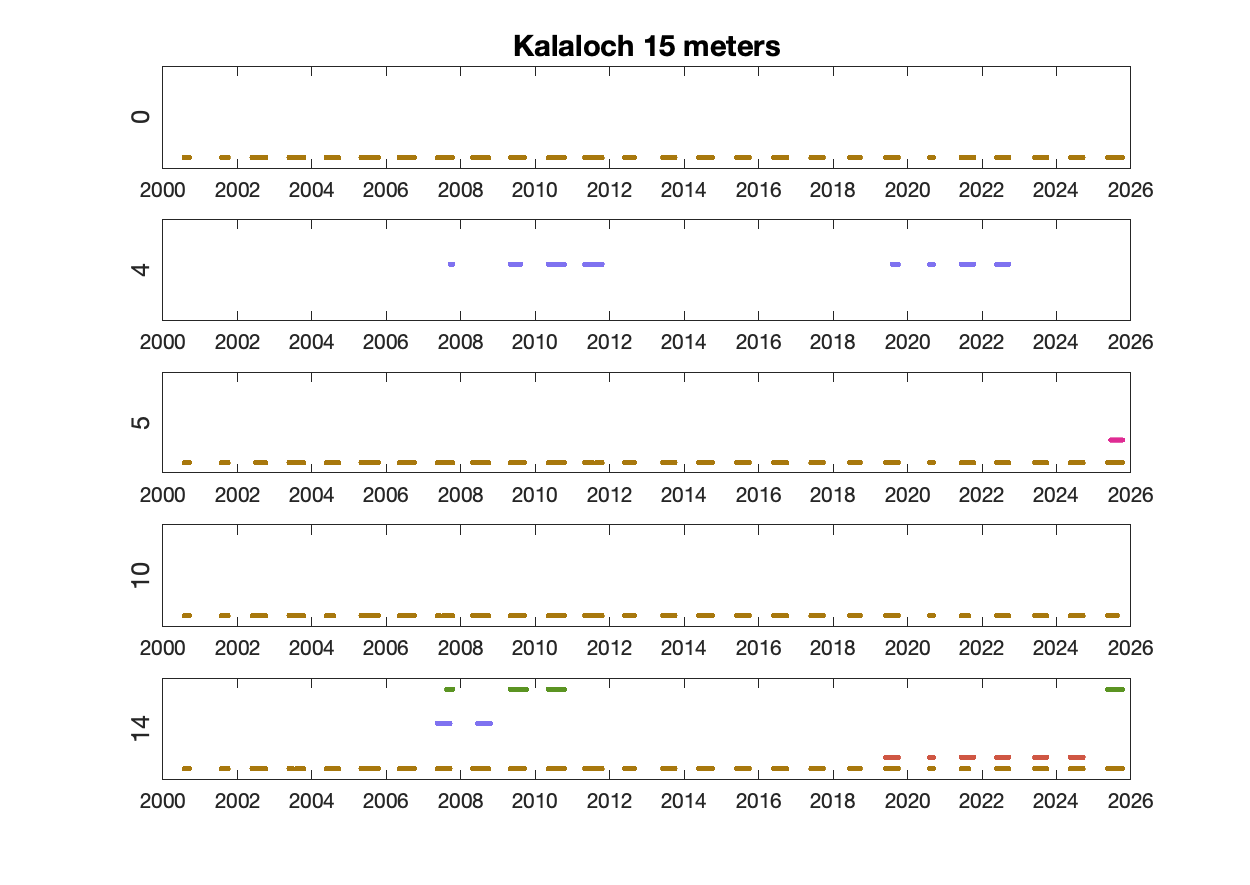


Figure S11. Timeline of measurements at the Kalaloch 15-meter isobath site.


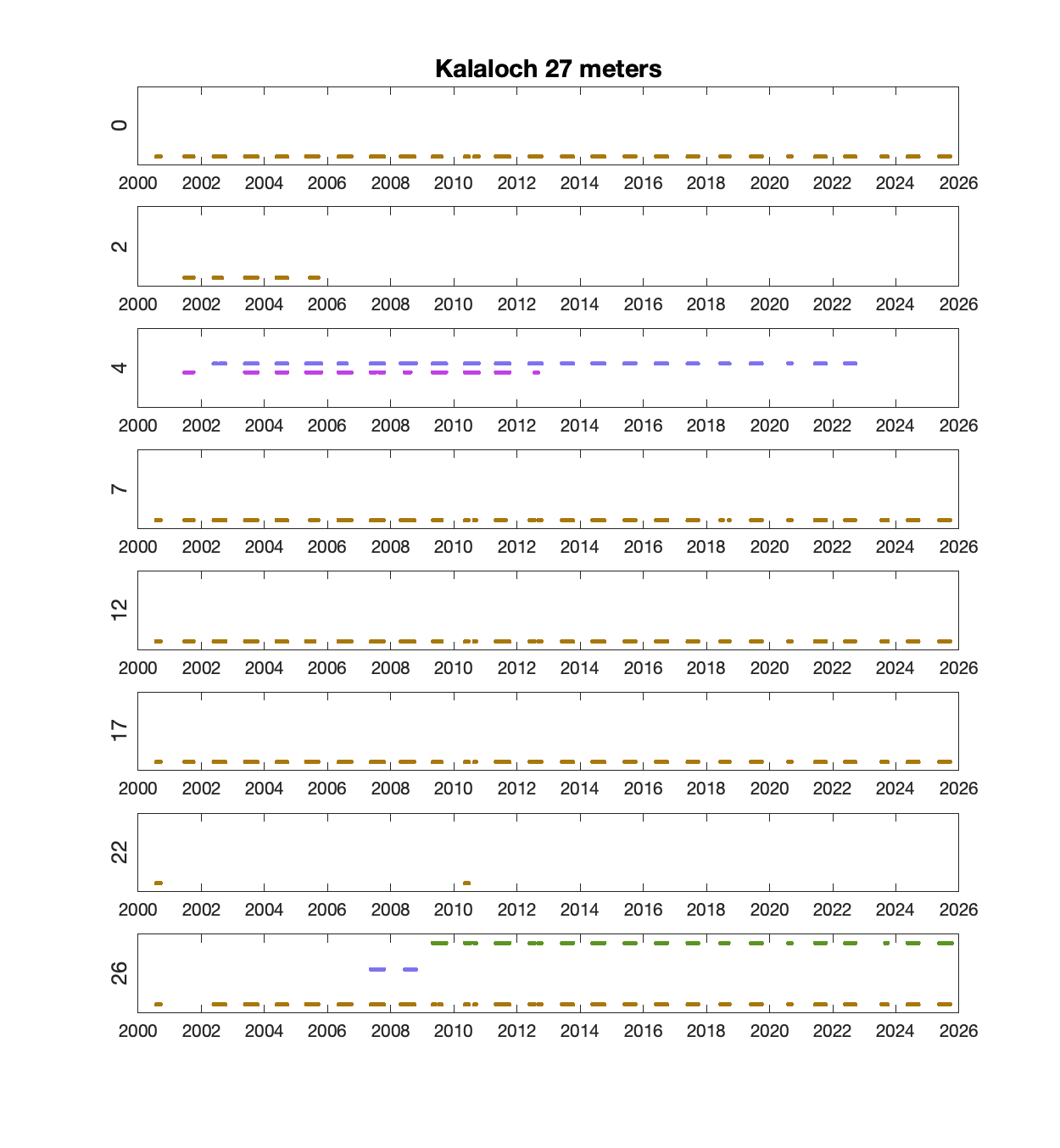


Figure S12. Timeline of measurements at the Kalaloch 42-meter isobath site.


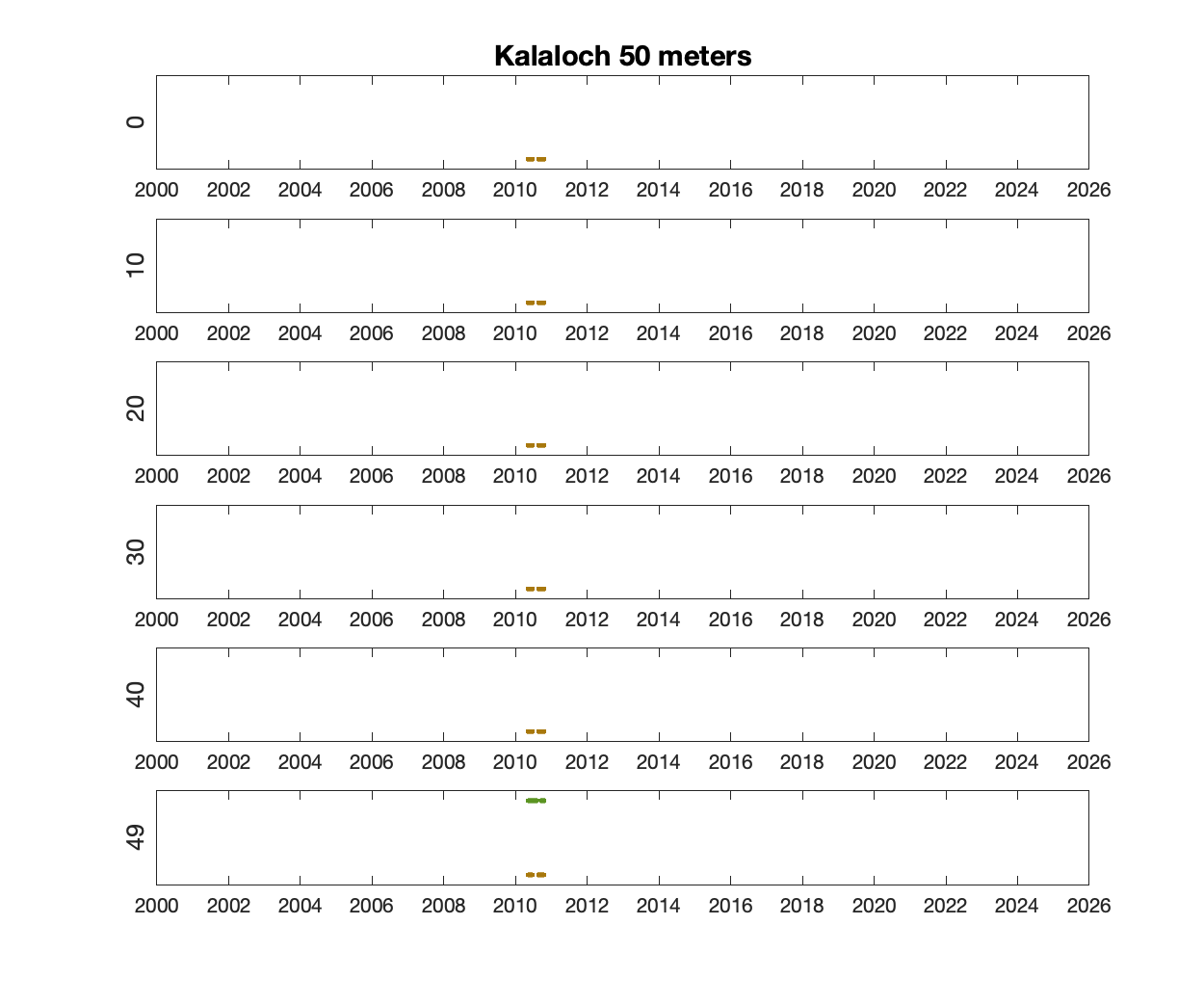


Figure S13. Timeline of measurements at the Kalaloch 50-meter isobath site.


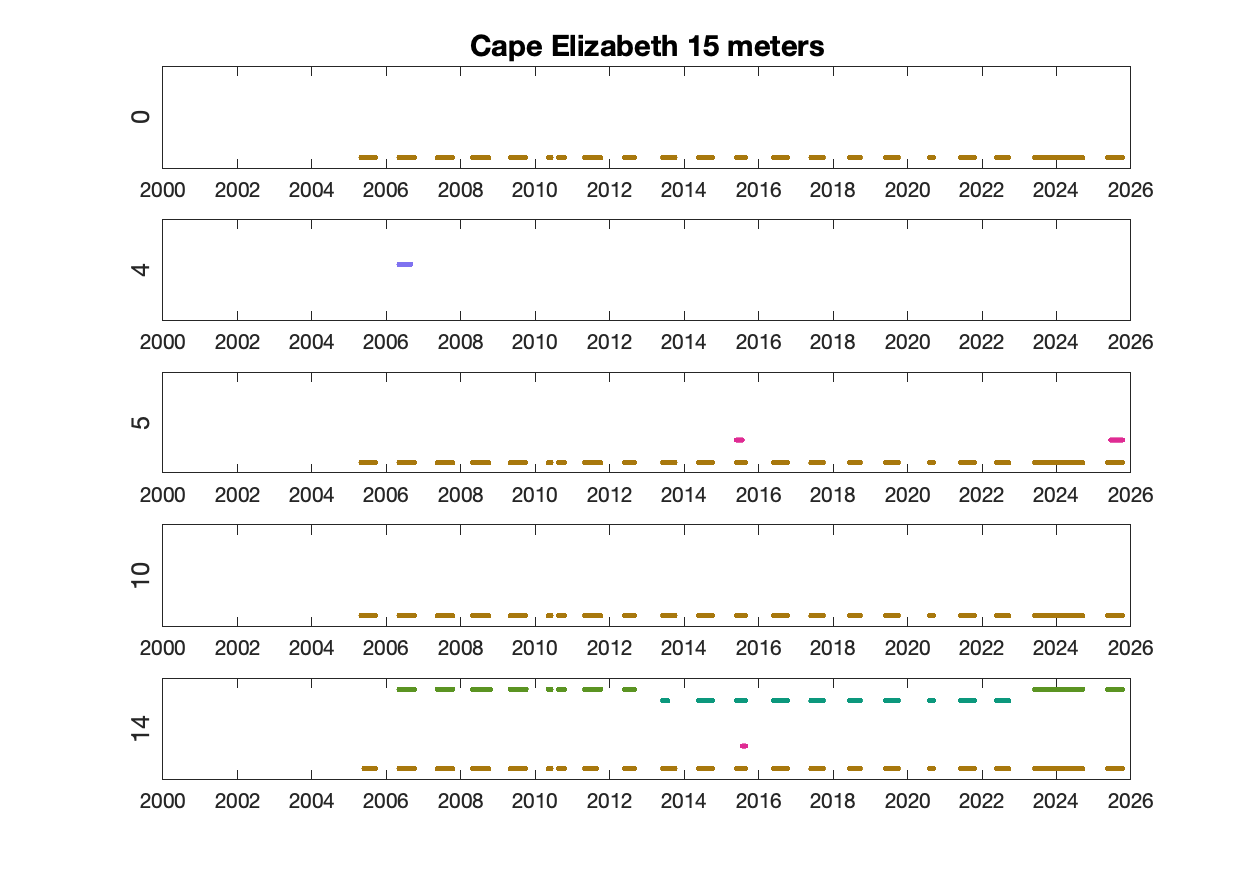


Figure S14. Timeline of measurements at the Cape Elizabeth 15-meter isobath site.


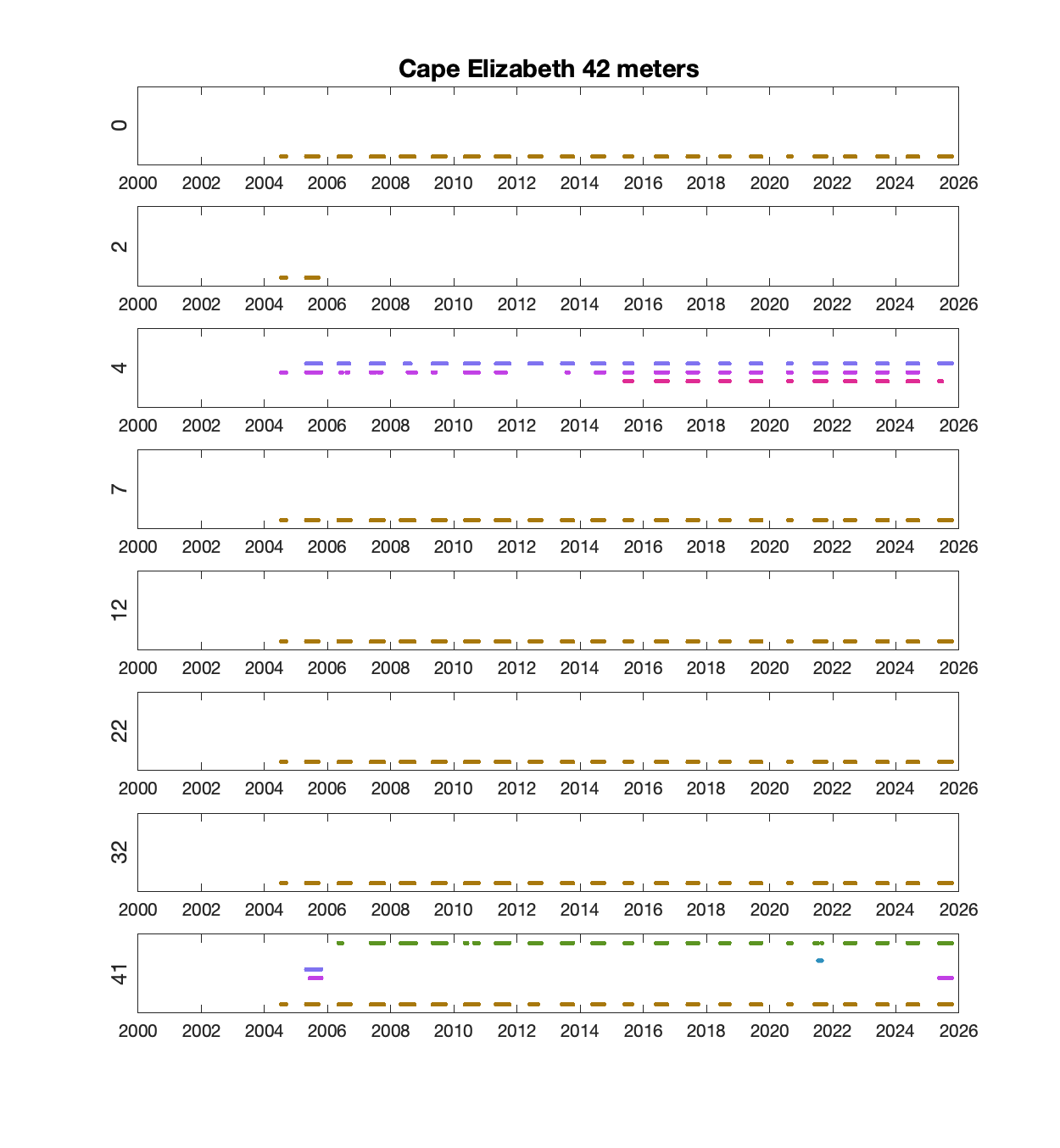


Figure S15. Timeline of measurements at the Cape Elizabeth 42-meter isobath site.


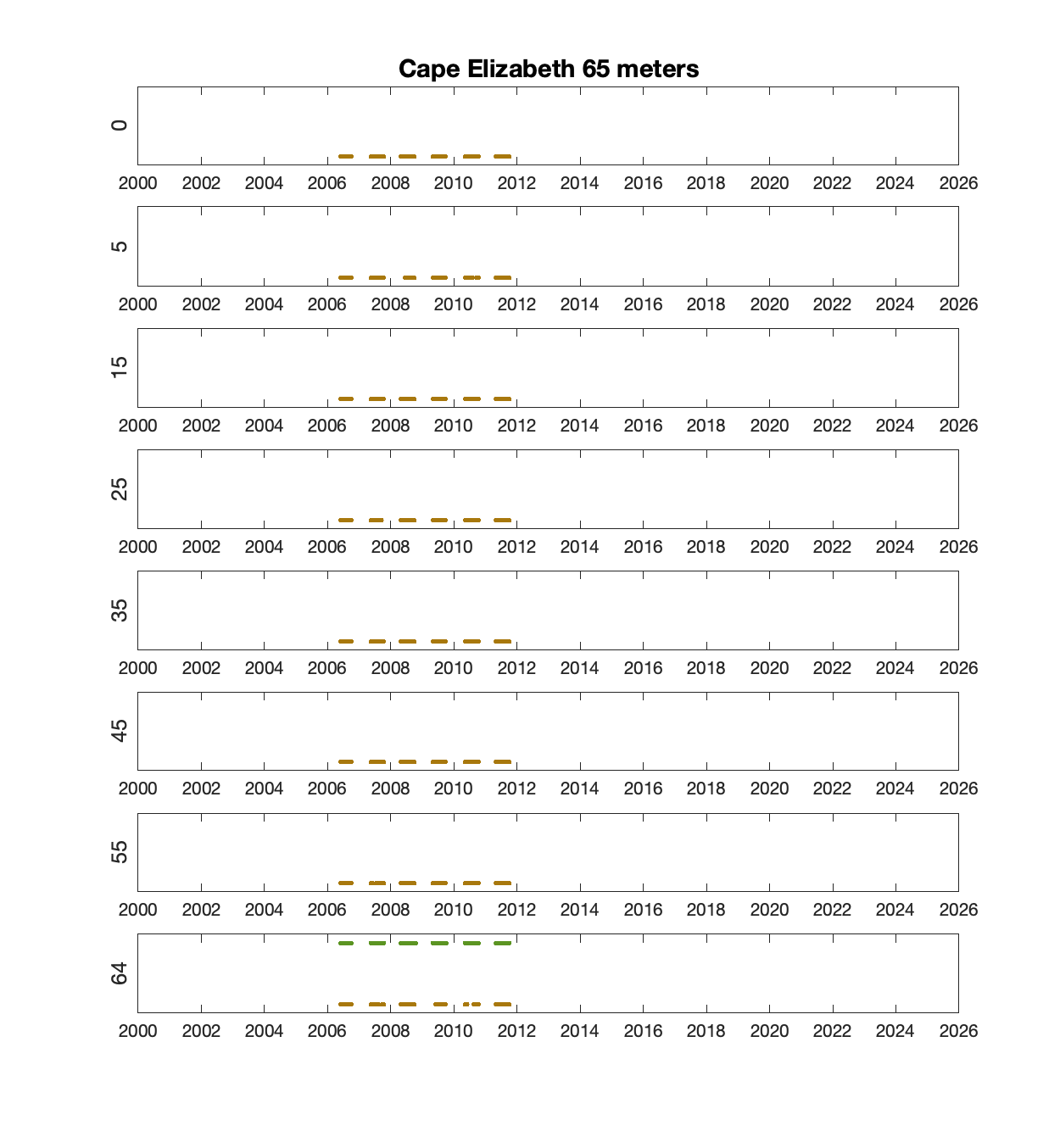


Figure S16. Timeline of measurements at the Cape Elizabeth 65-meter isobath site.
